# Process evaluation of a pilot multi-component physical activity intervention – active schools: Skelmersdale

**DOI:** 10.1186/s12889-018-6272-1

**Published:** 2018-12-18

**Authors:** Sarah L. Taylor, Robert J. Noonan, Zoe R. Knowles, Michael B. Owen, Stuart J. Fairclough

**Affiliations:** 1 Physical Activity and Health Research Group, Department of Sport and Physical Activity, Edge Hill University, St. Helens Road, Ormskirk, Lancashire L39 4QP UK; 2 Physical Activity Exchange, Research Institute for Sport and Exercise Sciences, Liverpool John Moores University, Liverpool, L3 2AT UK; 3 Department of Physical Education and Sports Science, University of Limerick, Limerick, Ireland

**Keywords:** Physical activity, Schools, Process evaluation, Children, Write draw show and tell, Teachers

## Abstract

**Background:**

Schools have been identified as key environments to promote child physical activity (PA). Implementation of multi-component PA interventions within schools is advocated but research has showed that they may not always be effective at increasing child PA. Results of the Active Schools: Skelmersdale (AS:Sk) multi-component pilot intervention indicated no significant positive change to child PA levels. Process evaluations can provide information on which aspects of an intervention were delivered and how. Therefore, the purpose of this study was to use a combination of methods to elicit child and teacher perceptions regarding the feasibility and acceptability of the AS:Sk intervention, alongside systematic researcher observations. The overarching study aim was to understand how schools implemented the AS:Sk intervention, with a specific focus on the frequency of intervention component implementation, and how the components were incorporated into the school day.

**Methods:**

The study generated five data sets. Data elicited from 18 participating children via a write draw, show and tell task included, frequency counts of most enjoyable intervention components, drawings, and verbatim data. Teacher verbatim data was collected from 3 interviews, and 18 researcher observations were recorded using field notes. The data sources were pooled to produce the themes presented in the results section.

**Results:**

The combination of data sources revealed four themes and 16 sub-themes. Implementation methods: how and when the components were implemented in schools. Child engagement: enjoyment and positive behaviour. Facilitators: peer influence, teacher influence, staggered implementation, incentives, rewards, challenges and competition, flexibility and adaptability, child ownership, routine.

Barriers: time within an intense curriculum, space, sustaining child interest, parental support, school policies.

**Conclusions:**

This study revealed that teachers believed classroom based activities were most feasible and acceptable due to the reduced implementation barriers of sufficient time and space. In contrast, children reported that the activities outside of the classroom were preferred. Future school-based PA interventions should aim to achieve a balance between routine PA at a set time and PA that is flexible and adaptable. Further process evaluations of multi-component school-based PA interventions are warranted to develop the limited evidence base.

## Background

Participation in physical activity (PA) during childhood, particularly moderate to vigorous intensity PA (MVPA), improves physical and psychosocial health [1–3]. National guidelines from across the world state that children and young people (CYP) should engage in 60 min of MVPA every day [4–7]. It is also recommended that children minimise time spent sedentary [4–7]. Engagement in sedentary behaviours is detrimental to many aspects of physical health such as body composition, cardiorespiratory fitness, metabolic syndrome, and cardiovascular disease risk factors [8]. A recent UK cohort study revealed that daily MVPA declines between the age of five and 9 years [9]. Sedentary time (ST) also increases during similar ages and overall, primary school children spend 55% of their time sedentary [10, 11]. This evidence warrants interventions which promote PA participation and ST reduction in children.

Child PA is a complex behaviour influenced by various individual, social and environmental factors [12]. Schools are key environments to promote PA regardless of children’s individual circumstances [13]. Furthermore, CYP spend a large proportion of waking hours at school (40–45%; up to 8 h). As such, child PA recommendations are increasingly being made in reference to school hours, with guidelines of children accomplishing 30 min MVPA within school [14, 15]. A range of PA interventions have been implemented in the school setting [16, 17], and there is consensus that multi-component interventions hold the most promise for increasing the PA levels of CYP [18]. However, multi-component interventions may not always be successful at increasing PA in CYP [19, 20], as demonstrated by our recent Active Schools: Skelmersdale (AS:Sk) pilot multi-component intervention, which did not result in significant changes in PA across the school day and whole weekday [21]. Multi-component interventions can be challenging to deliver, with inconsistent implementation (i.e., schools not implementing as intended) identified as a key barrier to success [19]. For example, the evaluation of the “Girls in Sport” multi-component school-based intervention focused on promoting PA among adolescent girls, found that only four of the 12 participating schools implemented the intervention as intended [19]. Whether school-based multi-component PA interventions succeed at positively impacting PA levels or not, it is important to understand how they have been implemented in practice, so that they can be further developed for future research and practice.

Process evaluations can provide information on which aspects of an intervention were delivered and how, which can allow for an accurate interpretation of either positive or negative outcomes [22]. Assessment of intervention implementation is also essential for understanding whether an intervention is internally and externally valid [22]. Although the process evaluation of interventions is an essential part of designing and testing complex interventions [23], there is a lack of evidence pertaining to both the quality and quantity of school-based PA intervention implementation [24].

UK Medical Research Council (MRC) guidelines suggest that both quantitative and qualitative methods have an important role in process evaluations, both independently and in combination [23]. Traditionally, school-based process evaluation studies have utilised methodologies such as teacher surveys, measures of child enjoyment, and registers of attendance [25–28]. However, methodological limitations are associated with some of these approaches. For example, the use of attendance registers may not always be appropriate for intervention components which are implemented within compulsory class time rather than optional extra-curricular PA. Furthermore, quantitative approaches provide limited information on the physical environment such as the setting within school which PA is taking place and also the social environment such as who children are taking part in PA with. Systematic observations of PA are said to be advantageous as they can provide such contextual understanding [28]. Observations of interventions in practice have therefore also been used in process evaluation studies [24, 28].

Qualitative methodologies allow for perceptions and experiences of interventions to be explored by those who have experienced them first hand. These approaches such as, teacher interviews, parent interviews, and child focus groups are subsequently the main components of school-based process evaluation studies [24–26, 28]. Focus groups have been deemed an appropriate and effective mechanism in children for collecting information with substantial depth and breadth [29]. However, some school-aged children in particular may struggle to verbalise their thoughts and/or feelings [30], and responses given can be brief and simplistic [29]. Hence, the use of a singular approach to explore children’s perceptions and lived experiences may not provide an accurate reflection of children’s intended meaning which subsequently limits understanding for researchers. A more developmentally appropriate and inclusive approach is to use focus groups in combination with drawings. Given that drawings are familiar classroom based activities this approach can provide children with greater control over their expression compared to verbal communication [31]. The combination of these approaches (focus group and drawings) through the write draw show and tell (WDST) methodology [32] can elicit a comprehensive and complimentary account of child perceptions and experiences of intervention programmes with a view to enhancing data credibility and trustworthiness [33].

The novelty of this process evaluation is elicited through capturing the perceptions and experiences of children via the developmentally appropriate and inclusive approach of WDST [32] and also the views of teachers via interviews. Conducting systematic observations supplements this qualitative data with information regarding the physical and social environment. The primary aim of this study was to understand how the participating schools implemented the AS:Sk intervention. This included the frequency of which intervention components were implemented and also how they were incorporated into the school day.

## Methods

Seven primary schools within Skelmersdale, a low-income town [34], within West Lancashire, UK, participated in the AS:Sk intervention. The methods and description of the AS:Sk intervention have been described elsewhere [21], but a brief summary is presented here. Following baseline data collection, four schools (*n* = 117 children) were randomly allocated to the intervention and the remaining three schools (*n* = 115 children) followed their usual practices as control schools. The intervention comprised of 8 PA components which were selected after consulting relevant school-based PA intervention literature and as a result of the findings from phase two of the AS:Sk project which piloted three components. The PA components also aligned with elements of the socio-ecological model [35], the Youth PA Promotion Model (YPAPM) [36], and the theory of expanded, extended, and enhanced opportunities for youth PA promotion [37]. Intervention approaches were designed to have minimal financial implementation costs to the project or schools (limited to printing costs). The PA intervention components included, active classroom breaks (ABs), Bounce At The Bell (a short, approximately 2 minute jumping routine to be performed before leaving the classroom for morning break, lunch break and before the end of the school day to introduce PA after typically long periods spent sedentary), ‘Born To Move’ (BTM) activity videos, Daily Mile (DM) or 100 Mile Club (MC) running/walking activities, playground activity challenge cards, PE teacher training (implemented online, all teachers received material via email including a presentation with audio, composed by the last author), newsletters, and PA homework. Schools were provided with a guidance document which provided specific detail on each intervention component. The first author’s email address and telephone number were also provided so that schools could seek additional information or support from the research team if required. Teachers also had opportunities to ask the first author any questions when visits were made to schools to conduct observations. All head teachers/principals of the intervention schools were aware of the intervention components. The level of head teacher/principal support provided to class teachers was not tracked as part of the process evaluation.

All four intervention schools were informed of the process evaluation requirements of the study before intervention implementation. Arrangements were made with relevant staff within each school for the procedures outlined in the section below to be completed during and after the implementation phase of the intervention. Due to class teacher absence and communication difficulties with the replacement teacher in one of the participating schools, only some of the procedures were completed. Ultimately, a decision was made to exclude this school from the current study due to the missing data sources which meant that a representative report on how and when intervention components were delivered could not be acquired. Subsequently, three schools were included in which all procedures were completed.

Year 5 classes (children aged 9–10 years) in the intervention and control schools participated in the study. Ethical approval was gained from the University Research Ethics Committee (ref #SPA-REC-2016-342). Passive (“opt-out”) parental consent were obtained in six of the schools, and one school chose to use active parental consent. All children completed informed assent forms prior to data collection, and teachers completed consent forms prior to participation.

### Procedures

#### Observations

The first author conducted observations and field notes for each intervention component. Observations were conducted at an agreed time with the class teacher. Where appropriate, for example with PE lesson observations, checklists were created based on the frameworks and principles with which the teacher training resources were based upon [38, 39]. A total of 18 observations were conducted throughout the intervention (six observations within each intervention school). The intervention components which were observed included, ABs (× 3), Bounce At The Bell (× 2), BTM videos (× 3), DM or 100 MC (× 4), playground recess period (× 3), PE lesson (× 3). The durations of these observations were sufficient enough to cover the whole period of time in which an intervention component was delivered. For example, for AB an observation would last for 5–10 min, while for a PE lesson an observation could last for up to 60 min. During all observations, field notes were collected in relation to aspects including the number of children involved, the location within the school, the behaviour and responses of children, and the actions of teachers.

#### Write, draw, show and tell groups

The WDST methodology was used with participating children to elicit their perceptions and experiences of the intervention components [32]. WDST and similar draw and write techniques have been used in previous child research to explore understanding of health and also more specifically, PA perceptions and experiences [32, 40, 41]. The WDST process has been described fully elsewhere [32, 40]. Its philosophical background is centred on a humanistic and ‘holistic’ approach in which the intervention implemented can be viewed through the eyes of the children rather than the eyes of the teacher or researcher [32]. This holistic approach is consistent with the more traditional focus group methodology.

A sub-sample of children from each school were included for the WDST methodology. No inclusion/exclusion criteria were set for children to be included, other than having parental consent to participate and that both girls and boys were selected, with the aim of an equal gender split. Once groups were arranged, they were conducted in a quiet area of the school whereby the researcher and participants could be overlooked but not overheard. Semi-structured WDST guides were developed and used to ensure consistency across WDST groups. Questions were informed by the YPAPM as this model provides a broad perspective on the factors that influence PA behaviour in children [36]. Questions also aligned to the overarching aim of the study which was to elicit children’s perceptions and lived experiences of the PA intervention components. The WDST guide was discussed with the second author, who developed the WDST methodology and has experience of qualitative research with children [32, 40]. It was agreed that the questions and tasks were developmentally appropriate and would enable the study aims to be achieved. Example questions used are displayed in Table 1, with an indication of their alignment to the YPAPM.

**Table 1:** Example WDST questions

YPAPM Factor	Intervention Component	Questions and Prompts
Predisposing – “Am I able” – *Perceived competence/self-efficacy*.	Active Breaks	Were the classroom based exercises hard or easy to complete?
		What was it about the exercises which made them easy/hard?
		Do you feel different doing the classroom based activities now compared to when you first started them?
		Can you think of how your body feels different?
Predisposing – “Is it worth it” – *Enjoyment*.	Born To Move videos	Was it enjoyable to take part in the videos?
		What did you enjoy about the videos?
		Was there anything you didn’t like about them?
Reinforcing – *Teacher Influence*	Daily Mile/100 Mile Club	What did your teacher do during the mile run?
		What is like to run the mile with your teacher taking part?

The WDST process began with the researcher presenting visual illustrations of each intervention component to children (e.g., an AB activity card which children would recognise from teachers using them with the class). This opening task was not to initiate any form of conversation but rather to allow children time to reflect on the intervention components. It also ensured that the children understood what the overarching topic of conversation was, so once questions were directed to specific intervention components children clearly understood what was being referred to. After children were shown the intervention component illustrations they were provided with a self-adhesive note-paper and were invited to write down which of the intervention components was their favourite, or they had found most enjoyable. Children were provided with the time to think about each intervention component, and then the opportunity to write down their favourite. Children then spoke aloud as they were asked to tell the group what they had written down as their favourite intervention component and explain why it was. This initial simple task helped build rapport between researcher and children and established an environment whereby sharing and listening was valued [32]. Once each child had contributed to the discussion, the session progressed with more challenging open-ended questions directed the whole group, taken from the semi-structured WDST guide.

To conclude the session, the draw aspect of WDST was used. Children were asked to independently draw a picture of themselves completing their favourite intervention component and to consider where they were in the school environment when doing so, who they were with and how they felt whilst taking part. They were also asked to summarise the picture with a short paragraph of words (write aspect) which would articulate the meaning embedded within their drawing. With the exception of providing children with motivational comments to continue/complete as appropriate, the first author refrained from providing any evaluation of the drawings [32]. Children were provided with the opportunity to again show and explain to the drawing to the group.

Three WDST groups were conducted in total (one per school), including 16 participating children (7 boys). Group sizes comprised four and six children, and the WDST sessions lasted 20–28 (mean = 24.7) minutes. All WDST groups were recorded using a digital recorder and were transcribed verbatim for further analysis and anonymised. This resulted in 61 pages of raw transcription data, Arial font, size 12, double spaced.

#### Teacher interview

Three class teachers were interviewed face-to-face by the first author. Semi-structured interview guides were developed and used to explore teachers’ perceptions and experiences of each intervention component with a view to understanding how they were implemented, the potential facilitators and barriers to implementation, and recommendations for future practice. Example interview questions included, “When have you been implementing active classroom breaks?”; “Do you think the activities are suitable for use in your classroom?”; “Are children motivated to participate in the daily mile?”; “What are the main barriers which have prevented you from using this component with your class?”; “What would your recommendations be to another teacher if they were planning on implementing similar PA strategies with their class?”. The interviews took place in a quiet, private area of the school at a convenient time for the teachers and lasted 15–28 (mean = 23) minutes. Teacher interview data consisted of 65 transcript pages raw transcription data, Arial font, size 12, double spaced.

### Data analysis

The study generated five separate data sources including child frequency counts of most enjoyable intervention components, child drawings, child verbatim data, teacher verbatim data, and researcher observations/field notes. Child and teacher verbatim data, and also the child drawings were analysed both inductively and deductively after the first author was familiar with the data (reading and re-reading of transcription text) [42]. Inductive analysis included producing initial codes and then searching for and reviewing themes before each final theme was clearly defined [42]. Themes were generated from the data in relation to the aims of the research without fitting them to a pre-existing coding frame [42]. As the YPAPM model [36] was used to underpin questions within the child WDST guides, this was also used for the deductive process as a thematic framework in the child verbatim data. For child drawings to be included, people, events, and/or places had to be recognisable. A ‘mark’ within a child’s drawing referred to an item which could be identifiable as a theme, the most basic example being other people drawn with the child indicating peers [32, 43]. Children’s narratives were transcribed verbatim, classified as a written ‘report’, and subsequently appended to each individual drawing.

The data sources (teacher transcriptions, child transcriptions, child drawings, observations) were pooled to produce the themes presented in the results section. This approach was taken for complimentary purposes, meaning that each separate data source could expand, enhance, and clarify the others [32]. This triangulation of methodology allows for cross-data validity checks between the child, teacher, and researcher [44]. Further review took the form of a presentation of the verbatim quotations and child drawings to the second author as a critical friend who had previously independently reviewed the data sources and cross-examined the data sources against the themes in reverse to offer alternative perspectives. Methodological triangulation and investigator critical review, combined with the use of verbatim transcription of data ensures methodological rigour, credibility and transferability [45, 46]. Where verbatim direct quotes are used, the data source, school, and gender of participants are outlined for clarification.

## Results

There were four themes generated from all data sources: implementation methods, child engagement, facilitators, and barriers. These four themes were then broken down further into more specific sub-themes. Themes are displayed in Table 2.

**Table 2:** Emerging themes from the data sources

Themes	Sub-theme
Implementation Methods	How
	When
Child engagement	Enjoyment
	Positive behaviour
Facilitators	Peer Influence
	Teacher Influence
	Staggered implementation
	Incentives, rewards, challenges and competition
	Flexibility and adaptability
	Child ownership
	Routine
Barriers	Time within an intense curriculum
	Space
	Sustaining child interest
	Parental support
	School policies

### Implementation methods

A summary of implementation methods across the three intervention schools is displayed in Table 3. Each intervention school was given freedom to implement the intervention components when it best suited their class and timetable. It was therefore important to summarise the differences between schools and understand how schools made the intervention components part of their working school day. This information was collected from researcher observations and teacher interviews. Implementation methods differed across each intervention school which limited the similarities observed between them. Similarities across the schools were to be expected because they all received the same content to deliver the components. For example, every school received the same health messages to be added to their newsletters, the same AB exercise cards to deliver PA in the classroom, and the same playground activity cards. Nevertheless, the frequency of newsletters for example differed within each school, and the way in which class teachers implemented the ABs differed within each school. School 1 used ABs to break up longer learning periods, School 2 implemented ABs at the start or end of lessons, whereas School 3 chose to display the AB exercise cards by the classroom door and complete the activities before morning break and lunch break as children lined up to leave the classroom. This latter approach was more similar to that recommended for the Bounce At The Bell component. With the playground activity cards, the specific implementation methods in School 3 led to the only engagement with the activity cards observed across the participating schools. School 1 did not use the cards at all, children did not engage in School 2 despite them being displayed, but in School 3 efforts by the playground staff to use music to engage the children in the activities were successful. These examples highlight how school differences influenced implementation methods and how adaptations to component protocols were made to suit each school’s own needs.

**Table 3:** Summary of implementation methods; how and when the participating intervention schools implemented intervention components

	School 1	School 2	School 3
Active classroom breaks	Used within longer morning or afternoon sessions to transition between tasks or break up tasks.	Implemented either at the beginning or end of a morning lesson, usually a maths or English lesson. Sometimes implemented immediately after returning to class from a morning assembly (which included 20–30 min of sitting, twice a week).	Three activity cards were chosen every morning and displayed by the classroom door. Activities were completed before morning break and lunch break as children lined up to leave the classroom. This was more of a Bounce At The Bell approach. Cards were sometimes used within lessons if children were getting restless or they needed a bit of a break.
Bounce At The bell	Deemed inappropriate as there were too many bells that go off in school for different class groups that can be heard by all.	Instead of using the school bell, the class teacher used an alarm sound from a phone which was played to initiate the jump routine. It was used predominantly in the afternoon when attention levels slipped.	Active classroom break cards used at break and lunch time ‘bell’.
Born to Move videos	Videos were used for a whole school ‘wake and shake’ on Tuesday and Friday mornings immediately after registration.	The class went to assembly 15 min early to complete a video. The children tried to complete videos within the classroom environment, but only certain aspects could be done.	This school had more control over their PE lessons and videos were therefore used in PE lessons as an active warm up. Also used in breakfast club (not all children).
Daily Mile/100 Mile Club	100 Mile Club implemented twice a week during two afternoons that an additional member of support staff joined the class. Children collected counters from a member of staff after each lap of the playground was completed. School staff calculated how many laps/counters was equal to a mile. Children completed their recording sheet once they returned to class, tallying their miles and counters. A classroom display board was made so children could see their own progression.	Children went out to the playground 15 min before lunch time to complete their Daily Mile, 12.00 pm. Afterwards they went straight into the dining hall to eat. Class teacher indicated that it wasn’t daily but rather three times a week at a minimum.	Daily Mile was implemented predominantly in the afternoon period. It was also integrated into PE and swimming (class walk to facilities). Class teacher indicated that it wasn’t daily, most commonly it was three times a week.
Playground activity cards	Activity cards not displayed.	Activity cards were tied to gates and fences around the playground. They began to look ‘scruffy’ after a few weeks because of weather conditions. Some initial engagement from children through curiosity but this wasn’t sustained.	Activity cards were displayed on the inside of a classroom window visible on the playground. A CD player was placed by the window outside where children could do the activities to popular music played out loud. It was predominantly girls that engaged in these activities
Enhanced PE	Limited attempts to decrease sedentary time. Static stretching, elimination within games and whole class feedback.	Some aspects of the SAAFE framework [38] and LET US Play [39] principles adhered to. Organisation of equipment allowed for an immediate start. Space was maximised for small group sizes. Limited teacher involvement.	Some aspects of the SAAFE framework [38] and LET US Play [39] principles adhered to. Warm ups were active with limited static stretching and the class was split into small groups. Sedentary time increased with whole group feedback and the organisation of equipment within the lesson.
Newsletters	Messages were included in 3 parent newsletters in total.	Messages were included in 3 parent newsletters in total.	Messages were included in 6 parent newsletters in total.
Physical activity homework	Attempts were made to hand out recording sheets on a weekly basis. Class teacher believed children lost interest after a few weeks due to having to repeatedly complete a daily recording sheet.	School employ a no homework policy. It was therefore implemented on a voluntary/optional basis and the teacher subsequently found it difficult to enforce. Not all children engaged.	School employ a no homework policy. It was therefore implemented on a voluntary/optional basis. A reward was handed out to the child who completed the most homework before the half term holiday.

### Child engagement

**Fig. 1:**
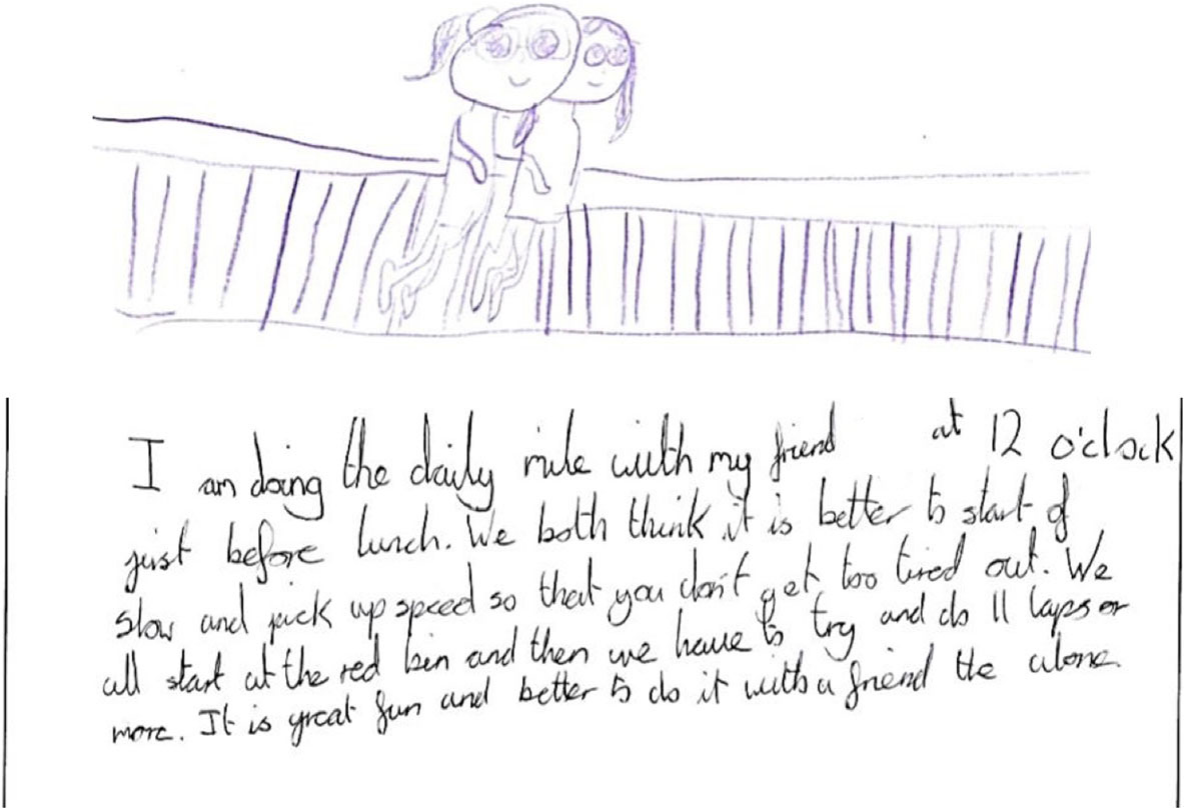
Drawing from a girl in School 2 illustrating the Daily Mile with her friend

**Fig. 2:**
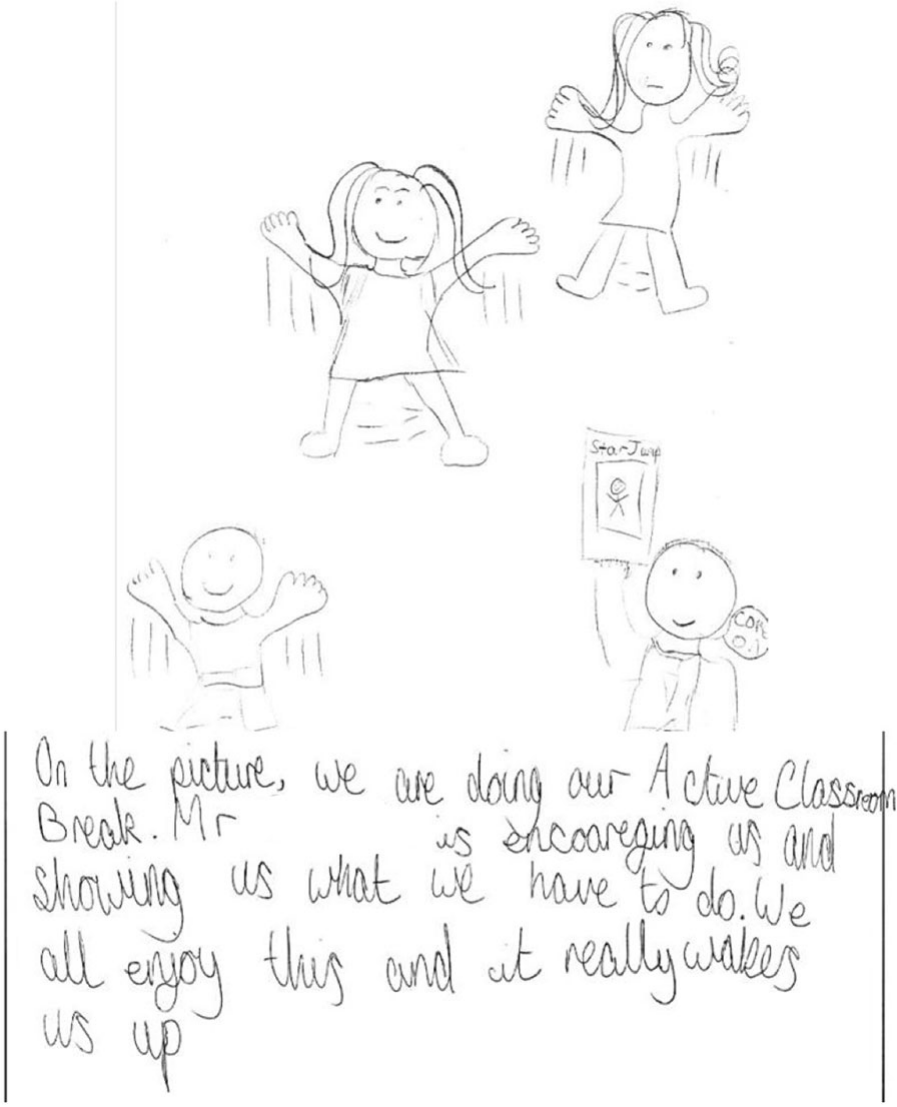
Drawing from a girl in School 2 illustrating taking part in active classroom breaks

There were two sub-themes relating to child engagement, which were enjoyment and positive behaviour. Enjoyment was consistent across all four data sources. The most enjoyable intervention components reported by the children were, the DM/100 MC (*n* = 11), BTM videos (*n* = 4), and ABs (*n* = 1). Positive behaviour was evident across all data sources apart from the researcher source. Evidence from each data source relating to these sub- themes are presented in Table 4. Figure 1 and Fig. 2 represent the WDST data sources for the sub-themes.

**Table 4:** Data sources for child engagement theme

	Teacher	Child	WDST	Observations
Enjoyment	“The more we got into it the more we enjoyed it. I thought it was absolutely great because it just gives the kids a bit of a break from learning and they loved it.” *S3, F (BTM).*	“They’re really fun because you don’t know what is coming next.” *S2, F (ABs).*	“I am doing the daily mile with my friend *child’s name* it is great fun”. *S2, F (DM,* Fig. 1*).*	There is good behaviour throughout all of the video, the teacher only speaks to encourage children. After the video finishes the teacher asks children to put their hand up if they enjoyed themselves, all children in the hall put their hands up in reply. *S3 (BTM)*.
Positive behaviour	“They settle back down onto task and they seem to be more settled and keen to start their work.” *S1, F (ABs).*	“Your brain is awake. We do the active classroom break and then we go back into maths, but you know more.” *S2, M (ABs).*	“On the picture, we are doing our active classroom break. We all enjoy this and it really wakes us up.” *S2, F (ABs,* Fig. 2*).*	

*F* Female*, M* Male

### Facilitators

**Fig. 3:**
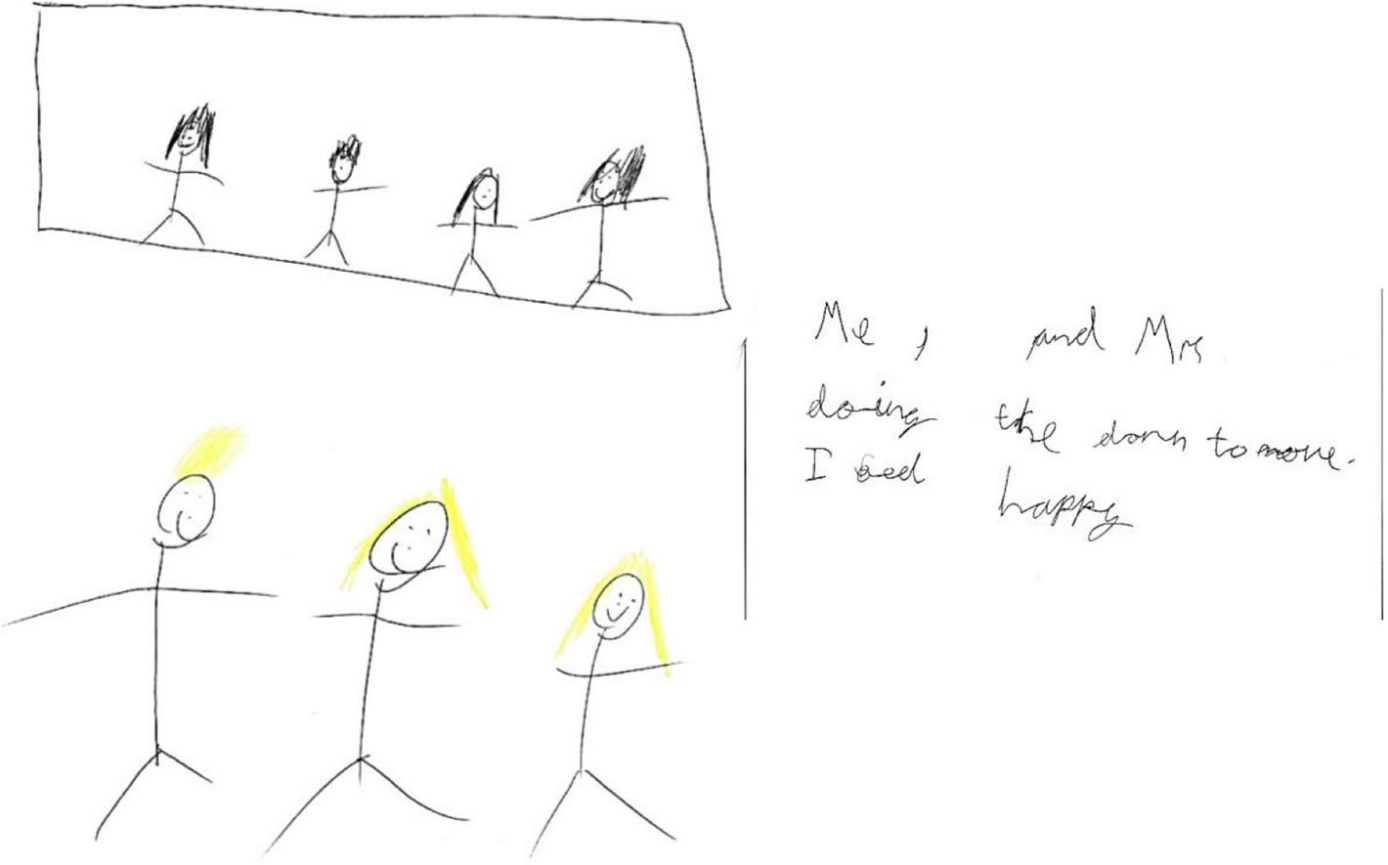
Drawing from a girl in School 3 illustrating taking part in Born to Move with her friends and teacher

**Fig. 4:**
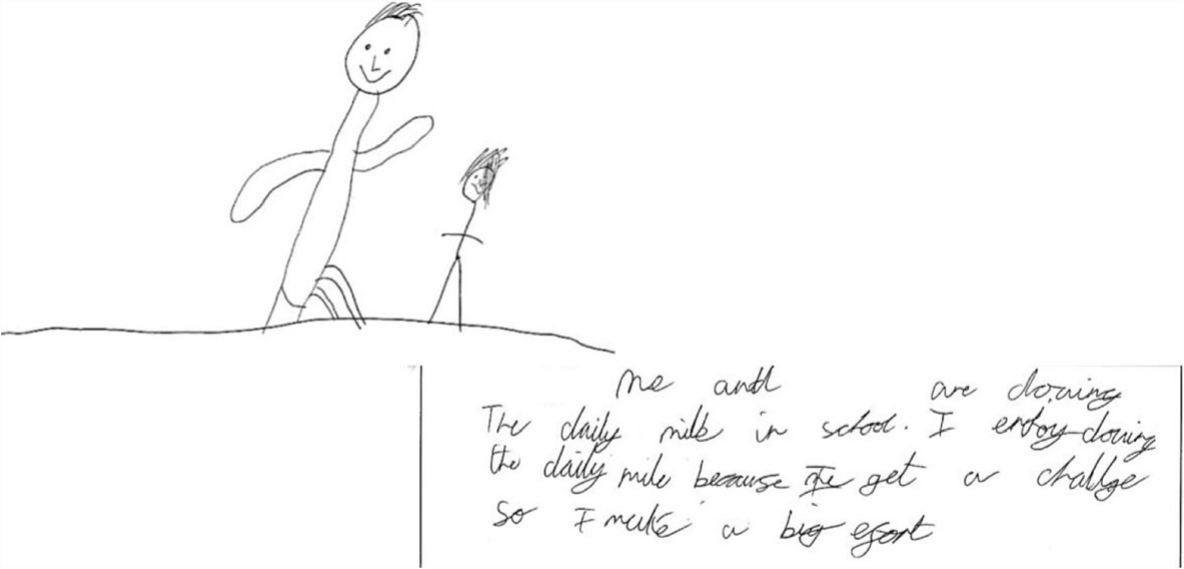
Drawing from a boy in School 3 illustrating taking part in the Daily Mile

There were seven sub-themes relating to implementation facilitators. Peer influence, teacher influence, incentives, rewards, challenges, competition, and routine were evident across all four data sources. A theme evident from teacher and child transcription but not child drawings was child ownership. Flexibility and adaptability was recognised by teachers, children and the researcher but was not evident in the children’s drawings. This is understandable as this concept may be more difficult to convey in drawing format. Only one theme came from teachers alone, which was staggered implementation. Evidence from each source relating to these themes are presented in Table 5. Figures 1, 3 and 4 represent the WDST data sources for the sub-themes.

**Table 5:** Data sources for facilitators to implementation theme

	Teacher	Child	WDST	Observations
Peer Influence	“It’s one of those where they get into groups and are like, right come on we’ll do this. With friends it helps.” *S1, F (100 MC).*	“It’s a bit more fun because you’ve got your friends with you to do it.” *S2, F (DM).*	“I’m doing the daily mile with my friend at twelve o’clock just before lunch. It is great fun and better to do with a friend than alone.” *S2,* F *(DM,* Fig. 1*).*	Children run in small groups of 2–3, talking and laughing whilst they move. *S2 (DM).*
Teacher Influence	“If I get involved with them they start to laugh at it because when I was getting it wrong or my teaching assistant was getting it wrong when we were doing it, it became comical because they’d teach me it.” *S2, M (ABs).*	“If the teacher is like, “you can do it”, and then we say, “ok, you do it” and then he’s like actually I can do it and it’s a bit more encouraging.” *S2, F (DM).*	*“*Me, *child’s name* and Mrs. *teacher’s name* doing the born to move. I feel happy.” *S3, F (BTM,* Fig. 3*).*	Throughout the video the class teacher is involved doing all the moves, when a child tells her they have a stitch she encourages them to carry on moving. *S3 (BTM).*
Staggered implementation	“Build it up gradually really, and sort of implement it a little bit at a time sort of thing.” *S1, F (General PA).*			
Incentives, rewards, challenges, and competition	“They quite like to choose their favourite video sometimes. So that is a bit of an incentive I use with them, that they can choose if they reach certain milestones.” *S1, F (BTM).*	“I like the Daily Mile because I can challenge myself to not stop.” *S3, F (DM).*	“Me and *child’s name* are doing the daily mile in school. I enjoy doing the daily because I get a challenge so I make a big effort.” *S3, M (DM,* Fig. 4*).*	In the classroom after the mile run children are excited to complete their recording sheet. Teacher calls out names and children shout out how many miles they are up to, the teacher gives praise and their names are moved up the miles on the classroom wall display. *S1 (100 MC)*
				After the run children tell their teacher how many laps they have done. One child doubled the amount of laps completed compared to the previous day and gets a round of applause from the class and will be pupil of the day the next day. *S2 (DM).*
		“We do it to beat *child’s name* because she’s the fastest in the class. She beat a teacher in a competition.” *S2, M (DM).*		
	“They go, “Right I’m going to try and do more than you”. So that positive competition was good for them.” *S2, M (DM).*	“I feel proud because I could have said, “No I don’t want to do it” but I did and I’m getting more done and I’ve got an extra mile.” *S1, F (100 MC).*		
Flexibility and adaptability	“Sometimes if they’re keen, they’re on with their work, I don’t stop them, but sometimes when they get to a point and you can tell they’ve reached that point of “I need to do something different”, then we do it”. *S1, F (ABs).*	“We put our own twists to the activities.” S2, F (ABs).		‘Shake it off’ exercise is the last to be performed in a 5 min active break. The teacher has speakers and ‘Shake it off’ song by Taylor Swift ready to play. Children sing along and get a boost from the music to put greater effort in. *S2 (AB).*
	“They ended up loving head, shoulder, knees and toes, so we did that in several languages as we went through. Luckily my teaching assistant speaks multiple languages so that became comical.” *S2, M (ABs).*			
Child ownership	“Sometimes if we’re busy with a child, explaining a concept or something, some of the kids will just take the lead and they will let the whole group do it”. *S3, F (Bounce at the bell).*	“When *child’s name* gets to pick which one, he’s super enthusiastic.” *S2, F (ABs).*		
Routine	“It became much easier, particularly with the mile a day. That was easy to be able to do. You know, quarter to twelve, twelve o’clock every day because that was just before lunch.” *S2, M (DM).*	“You have English for an hour and you go out at twelve o’clock and just do it.” *S2, M (DM).*	“I’m doing the daily mile with my friend at twelve o’clock just before lunch.” *S2,* F *(DM,* Fig. 1*).*	Children start to get ready to go outside after a class test at 11.58 am, by 12 pm children are on the playground running their mile. Once the run has finished after 15 min, children eating hot food from the school kitchen go straight into the dinner hall, children with packed lunches go back to the class room to get their food. Children didn’t need direction or instructions after the run as to what to do next. *S2 (DM).*
	“In the end it became much easier because it just became routine to have three, four things happening during the day most days.” *S2, M (General PA).*			

*F* Female, *M* Male

### 7.3.4 Barriers

There are five sub-themes relating to implementation barriers, these were time within an intense curriculum, school space for activities to be performed, sustaining child interest, parental support, and school policies. Sustaining child interest and parental support referred to the PA homework component only and was consistent between teacher and child verbatim data. Both teacher and child verbatim data also highlighted the barrier of time especially within the intense curriculum. Space and school policies came from the teacher data source only. Researcher observations did not highlight any of these barriers most likely because they were conducted through pre-arranged visits. Evidence from each source relating to these themes is presented in Table 6.

**Table 6:** Data sources for barriers to implementation theme

	Teacher	Child	WDST	Observations
Time within an intense curriculum	“There are times where activity will just get knocked on the head, because I’ve got to fit everything in before Christmas, otherwise I’m going to be behind for testing at the end of the year. Next half term it’s pretty much maths and English and reading for them they don’t really have the creative side, the exercise side, the fun bit of education because it’s all test-based.” *S2, M (General PA).*	“In the assessments, when we were doing our last test we couldn’t do it.” *S2, F (ABs).*		
School space for activities to be performed	“We’ve only got one hall slot per year group per week.” *S3, F (BTM).*			
Sustaining child interest	“They liked doing it initially but then they found after about four or five weeks, because it was repetitive, the same thing, they thought, “Oh Miss, it was getting a bit of a tedious task”, and the number of them completing it dwindled.” *S1, F (PA homework).*	“Sometimes you can forget to fill the sheet in.” *S1, F (PA homework)*		
Parental support	“A lot of the parents are at work so some of them say to you it’s even hard for them to sign a record for the kids this age so the lads do it themselves, but then you haven’t got parental support because the children are actually managing their own, so it’s a really big grey area and I’m assuming that happens in all schools.” *S2, M (PA homework).*	“My Dad is at work from very early in the morning to late at night.” *S1, F (PA homework).*		
School policies	“I couldn’t enforce it because we’ve sent a letter out to parents to say there’s going to be no homework other than learning spellings.”			
	“I couldn’t enforce it because we’ve sent a letter out to parents to say there’s going to be no homework other than learning spellings.” *S2, M (PA homework).*			

*F* Female, *M* Male

## Discussion

This study aimed to combine the qualitative data collected from children, teachers and researcher observations within participating AS:Sk intervention schools to assess the implementation of multiple PA components within the school day. Four themes emerged from the data. Implementation methods included sub-themes of how and when teachers implemented components. Child engagement with the intervention included sub-themes of enjoyment and positive behaviour. Another theme was the facilitators to implementation which included peer influence, teacher influence, staggered implementation, incentives, rewards, challenges and competition, flexibility and adaptability, child ownership, and routine sub-themes. Finally, barriers to implementation included sub-themes of time within an intense curriculum, space, sustaining interest, parental support, and school policies.

### Implementation methods

ABs were most commonly implemented at the start or end of a lesson. Teachers also deemed ABs to be useful for ‘breaking up’ a lesson or transitioning from one subject to another. The use of ABs at the end of an academic lesson has been reported by other teachers who implemented them as a reward for children after academic engagement [47]. One school used the AB activity cards combined with the Bounce At The Bell component, i.e. three AB activity cards were completed before the start of morning and lunch break (instead of the prescribed jumping routine for Bounce At The Bell). Bounce At The Bell in its prescribed format was not implemented by any schools. Conversely, in a previous Bounce At The Bell intervention study it was deemed to be an exercise programme that could be easily implemented and compliance to 10 jumps completed three times a day, ranged from 2 days to 5 days per week [48]. Bounce At The Bell was adapted in one school to be used during lessons to get children’s attention with a teacher-implemented bell noise that initiated a jumping routine, usually once per afternoon.

BTM videos were successfully implemented twice per week in one school with a whole-school (all age groups) approach, and were used within PE lessons in another school. BTM videos were prescribed to complement rather than replace PE, although this particular school chose not to implement outside of PE time. Given that the videos were 10 min long, this school found it appropriate to use the videos as an active warm up within the start of the PE lesson before moving on to taught content. Two schools implemented the DM, although both struggled for this to happen every day. One school had a set time every day (just before lunch break) but the other implemented whenever necessary, which was commonly in the afternoon. The remaining school chose to implement the 100 MC. This occurred twice a week and the number of laps needed for a mile were calculated, with children collecting a counter after every playground lap completed. Individual child records or laps and miles completed were kept and updated after every run.

Playground activity card engagement was limited and the inclement weather conditions caused the quality of the cards to deteriorate. This component was most successful in a school that displayed the cards on the inside of classroom window with music playing. Within PE lessons there was some evidence of attempts to decrease stationary and sedentary time (i.e., teachers complied with some components of the checklists that were created based on the LET US PLAY framework [39] and SAAFE principles [38] on which the teacher training resources were based). However, overall this evidence was limited and many of the principles were not implemented. In a study including a full-day professional learning workshop for teachers, more systematic observations indicated that teachers adhered to between 62.9 and 79% of recommended PE lesson structures or techniques to enhance MVPA [49]. Teachers found the PA homework difficult to sustain due to interest levels of children which reduced with time. Finally, all schools posted PA messages within their newsletters (minimum of three messages) which were circulated to parents. Overall, none of the schools implemented the multi-component intervention consistently with its prescribed format. There was evidence of reduced implementation compared to frequency guidelines. Also adaptations compared to delivery guidelines were evident to suit the individual needs of each school.

### Child engagement

The sub-themes of enjoyment and positive behaviour within the child engagement theme are consistent with other school-based PA interventions [50–52]. Fun and enjoyment in particular have been outlined as key areas of focus for PA promotion in young people and is a predisposing factor for PA participation in the YPAPM [36, 53]. Enjoyment is a stable and consistent psychological construct which predicts PA participation, and PA participation has been shown to have a positive effect on academic behaviours, including better attention and on-task behaviour [54, 55]. As child enjoyment and positive behaviour are recognised as benefits of school-based PA, they should be used as key drivers to help engage head teachers and class teachers in future school-based PA interventions.

### Facilitators to implementation

One participating intervention school consistently implemented BTM videos twice a week. This was facilitated by implementation occurring with the whole-school (all age groups) with completion of an active “wake and shake” together at the same time. This was the only evidence of a whole-school approach across the participating intervention schools and intervention components. Although having all children participating at the same time may not be possible for larger schools, where feasible, this approach should be considered in future school-based PA interventions. Rather than competing for school space, teachers were able to use the space together with a shared vision for increasing PA, and this supportive school climate has been identified as a key factor in a systematic review of implementation literature [24]. Head teachers have also previously stated that activities including the whole school make it easier to manage effectively within a school environment [56].

Teachers also referred to routine as a facilitator to implementation. Teachers believed that eventually the intervention components became part of their routine, and this was facilitated by a staggered implementation of components so not to initially overload the timetable or children with activity. Additionally, in School 2 for example, having a routine with the DM and consistently completing it at the same time of the school day aided implementation. That being said, teachers reported that flexibility to implement whenever best suited the class was also important, which has also been reported as a facilitator in previous studies [24]. Head teachers have also stated that interventions with a flexible approach are useful within primary schools [56]. Furthermore, teachers adapted intervention components from their prescribed format to also best suit their class, for example the ABs and Bounce At The Bell were implemented differently across schools, at different times of the day and implemented in different ways. School 2 would implement ABs at the start of a lesson whereas School 3 used AB cards at bell time (the end of lesson time). Previously, ineffective outcomes have been associated with programmes not being implemented as intended [57]. However, the school setting is dynamic in nature with constant change to schedules for example, and researchers should consider this when designing and evaluating school-based PA interventions. If schools are compromising on intervention fidelity this may not always be a failure of the intervention. For example, if changes are made by schools to intervention prescription in order to make strategies work best for their individual circumstances, PA could be more likely to occur within the school day. This flexibility and adaptability were reflected in reports of adding incentives, rewards, challenges, competition, and child ownership which all facilitated implementation and child engagement.

Two reinforcing factors of the YPAPM model were consistently reported by both children and teachers alike; these were peer and teacher influences [36]. Teachers recognised that children preferred to do PA with their friends, and children echoed these thoughts by stating how activities were more fun with their friends taking part with them. Previous research has indicated that friends’ PA levels can have a significant influence on an individual’s PA level and has recommended that future PA interventions consider encouraging friends to be active together [58, 59]. Teacher involvement included verbal encouragement and also taking part in some of the activities, with children recognising the encouragement in particular to make them try harder. The pivotal role which teachers play was highlighted by the Fit-4-Fun study which resulted in increased PA levels which were mediated by teacher support [60].

### Barriers to implementation

Five sub-themes were reported by teachers in relation to barriers which prevented implementation and subsequently will have impacted the efficacy of the AS:Sk intervention. Time within a crowded curriculum was cited by teachers as a reason for having to omit intervention components and PA in general, within the school day. Children were also aware of time pressures particularly when they had academic assessments to complete. The barrier of time is consistent with previous school-based intervention implementation literature [24, 61, 62]. High intervention fidelity was reported in a healthy lifestyles programme to prevent obesity, as education intervention components were compatible with the National Curriculum and did not displace teaching time [28]. For PA components (rather than educational components) this may be a more difficult task, although the integration of movement with academic outcomes has recently received more attention in the literature [63, 64]. Despite this active learning approach, teachers have still reported time as a barrier to the delivery and implementation of such movement integration strategies [65]. Physically active lessons are often short-lived and are not sustained in the long-term [66]. If this approach to PA promotion is considered by schools or researchers designing future school-based interventions, attention should be given to the barriers which impact teachers’ ability and motivation to implement physically active lessons, from individual, interpersonal, institutional, and community level perspectives [66]. Furthermore, both teachers and children alike spoke positively about ABs as a means for a short break from academic content. Teachers have previously reported that ABs can be fitted into schedules without disrupting academic lessons [67]. When considering the barrier of time within classrooms for PA strategies, ABs may not always be viewed negatively as an activity which takes time away from the curriculum by teachers.

Although time was cited as a barrier for the intervention as a whole, more commonly teachers referred to time barriers when discussing intervention components that were required to be implemented outside of the classroom, for example completing a mile run or an active video. This suggested that ‘transfer time’ to different locations within school would add significantly to the amount of time needed and subsequent perceived teacher burden. These intervention components were also limited by the barrier of school space. Limited school space is something that has previously been observed in school-based PA interventions [26]. Teachers reported attempting to complete BTM videos within the classroom due to not being able to access the hall/gym, and overall it appears that classroom based activities increase feasibility of implementation. Although, when considering the intervention components children reported as being most enjoyable, the two most common were activities outside of the classroom (DM/100 MC and BTM videos). Thus, within the school environment a compromise between child enjoyment and teacher practicalities is evident and the importance of gaining perceptions from both children and teachers is highlighted. Whilst children may find activities outside of the classroom in a different environment more enjoyable to participate in, teachers face more difficulties in trying to implement these types of activities. As teachers found classroom-based activities more feasible to implement compared to those implemented outside of the classroom this may explain some of the PA results of the AS:Sk intervention. It is difficult to fully explain why the AS:Sk intervention reduced ST but did not increase MVPA [21], but there was less guarantee that intervention strategies would stimulate PA of sufficient intensity to increase MVPA. Stimulating PA of a moderate or vigorous intensity is dependent on factors such as fitness, motivation, time and space. In contrast, decreasing ST is arguably less dependent on these factors which are more influential on MVPA engagement. Space in particular would have been limited within classroom-based activities which appeared most feasible for teachers to implement within AS:Sk.

A lack of parental support was reported by both teachers and children as a barrier to engagement with the PA homework intervention component. The PA outcomes of the AS:Sk intervention revealed that there were no significant effects on whole weekday movement behaviours (including out of school hours) [21]. Within the YPAPM, family influence is stated as a reinforcing determinant of PA engagement [36]. Thus, the lack of parental or family support reported may explain why the AS:Sk intervention was not effective in increasing whole day PA. Additionally, it is likely that the PA homework and newsletter components were insufficient to engage children and families and lead to meaningful changes in activity behaviours. A suggestion based upon these results was that more substantial efforts to promote engagement in PA during out of school hours are needed [21]. For example, with school-based extracurricular PA opportunities, rather than PA that requires children to engage within the home environment [21].

### Strengths and limitations

The main strength of this study is the multiple sources from which data were collected. Perspectives from teachers via interviews, children via WDST/focus groups, and the researcher via observations, helped to provide a comprehensive picture of the intervention and how it was implemented. Furthermore, the triangulation of data methodology enhances credibility. However, given that PE teachers were involved in the AS:Sk intervention via the online training component, not collecting their perspectives via interviews is a limitation of the study. The small number of participating schools is also a study limitation. This number was reduced further due to missing data and consequent exclusion of one of the original intervention schools. Although the circumstances of this school were unique, with staffing changes mid-intervention, increased communication between teachers and researchers may have reduced the likelihood of missing data which resulted from the situation. In practice though, it was difficult for the researcher to intervene, which reflects just one of the many complications which researchers can face when collecting data in school settings.

A further limitation is the lack of quantitative data collected. Resultantly, there is a limited understanding on specifically how often each intervention component was implemented week by week. Although teachers indicated how often components were implemented, this relied on teacher-recall over an eight-week period to provide a general picture of how the intervention was implemented, rather than specific frequencies. Initially, recording sheets were given to teachers for the 8 weeks of the intervention period and teachers were reminded to complete these sheets to indicate how often they had implemented each component per day. However, teachers reported that they would often forget to complete and/or lose recording sheets. Teachers most commonly ‘forgot’ because of the limited time they had to complete other teacher related administrative tasks, again highlighting the difficulties of school-based research. In a previous school-based intervention that used weekly log sheets, adherence of completion was low (34% of eligible weeks) [62]. Whilst attendance records are a feasible method for measuring uptake to after-school clubs or workshops for example [28], greater consideration is needed for more feasible methods for the tracking of school-based PA interventions throughout the implementation period, which are not reliant on teacher logs or daily researcher visits. Stronger teacher engagement in the implementation process could be achieved whereby teachers adopt a research role within projects. Teachers would subsequently engage in professional development and this approach would allow for schools to have greater ownership over their involvement in research projects and the data that is produced. ‘Teacher as researcher’ has previously been proposed as a way to develop teaching techniques but is yet to be used within school-based PA research [68, 69]. However, this approach would not remove teacher burden. Thought is needed for how research involvement for teachers could be integrated into their busy schedules and what is already a multi-role profession.

## Conclusions

The process evaluation of the AS:Sk intervention demonstrates that time is a key perceived barrier to PA among teachers and children. Because of limited time and space, classroom based activities may be most feasible and acceptable for teachers to implement, although children reported that the activities outside of the classroom were most enjoyable. These findings together highlight the compromise required within school-based PA interventions to accommodate components which *children will want to participate in* and which *teachers can feasibly implement* within the school day. In order to understand whether strategies are both enjoyable for children and feasible for teachers, capturing the voices of children and teachers is essential. Also, a whole-school approach with teachers working together with multiple year groups to implement PA can remove the barrier of competing for space. However, schools may struggle in finding complimentary times between different timetables. Future school-based PA interventions should aim to achieve a balance between PA being implemented at consistent specific time points in the school day, whilst also having capacity for PA components to be flexible and adaptable so that they can suit the individual needs of specific classes. Enjoyment of PA and the positive effect it can have on behaviour are important ‘selling points’ to schools and school staff to encourage future participation in school-based PA interventions and overall engagement in school-based PA.

## Abbreviations

100MC: 100 Mile Club
AB: Active classroom break
AS:Sk: Active Schools: Skelmersdale
BTM: Born To Move
CYP: Children and young people
DM: Daily Mile
F: Female
M: Male
MRC: Medical Research Council
MVPA: Moderate to vigorous physical activity
PA: Physical activity
ST: Sedentary time
WDST: Write draw show and tell
YPAPM: Youth physical activity promotion model
